# The Best Practice Conference: An Interactive Practice-Based Learning Activity for Resident and Faculty Development

**DOI:** 10.15694/mep.2019.000022.1

**Published:** 2019-01-29

**Authors:** Randon Welton, Tana Andre

**Affiliations:** 1 Wright State University

**Keywords:** Practice-based learning, Peer-based learning, Continuing Medical Education, Flipped-classroom

## Abstract

This article was migrated. The article was marked as recommended.

**Objective:** The Best Practice Conference (BPC) was designed to use active techniques to enhance measurable learning from a continuing medical education program.

**Design:** The BPC incorporated flipped-classroom techniques, guided discussion, and peer based components to enhance learning. The impact of the BPC was measured by comparing pre-conference, post-conference, and 3-month follow-up data.

**Setting:** The BPC started with emails to likely attendees. The conference occurred during a regularly scheduled Grand Rounds time slot. There were subsequent follow up e-mails to those who had attended.

**Methods:** The Best Practice Conference involved e-mail contact with residents and faculty members who were likely to attend the conference. These e-mails included clinical scenarios, questions about treatment options, and journal articles. The BPC lasted for 1.5 hours and included a variety of active learning techniques. These conferences were attended by psychiatry residents and faculty members. The attendees were contacted several months later and asked questions about the BPC.

**Results:** Between 39 - 48% of attendees changed their recommended treatment over the course of the conference, and three months later approximately 10% of attendees reported that their practice patterns had changed because of the Best Practice Conference. The Best Practice Conference catalyzes developmental learning where clinicians critically evaluate their practice habits in the context of the medical literature and the practices of their peers.

**Conclusions:** The Best Practice Conference promotes impactful Practice Based Learning and Improvement among residents and faculty members.

## Introduction

The Accreditation Council for Graduate Medical Education enshrined Practice Based Learning and Improvement (PBLI) as one of the core competencies. “Residents must demonstrate the ability to investigate and evaluate their care of patients, to appraise and assimilate scientific evidence, and to continuously improve patient care based on constant self-evaluation and life-long learning” ( ACGME, 2017). Ideally this practice continues throughout their career. Meaningful PBLI includes a self-examination of individual practice patterns and patient outcomes and a literature review to ascertain current recommendations and expected patient outcomes. Discrepancies may indicate a need for change ( Burke et al., 2014). Promoting PBLI becomes challenging with residencies’ faculty members. Access to provider data and patient outcomes and the ability to influence provider practice may be absent in decentralized, community programs.

Many traditional methods for educating physicians have little evidence of success in improving practice or patient outcomes. A review of 61 educational interventions in 31 studies found that the averaged effect size on physician performance and patient outcomes was small (0.18 and 0.14) ( Mansouri and Lockyer, 2007). Didactic conferences or distributing printed information may increase knowledge, but do not seem to significantly impact physician performance. Interactive strategies including small group interactions and case discussions have the best chance at inducing changes among providers ( Mansouri and Lockyer, 2007; Bloom, 2005; Davis, 1998).

Problem-based learning often entails assigning readings to students prior to the training experience. The group engages in an in-depth discussion of a specific case in light of the distributed material. Considering the information in a clinical context makes it more transferable to existing patients. Problem-based learning seems to be somewhat effective in changing doctor performance and patient health ( Al-Azri and Ratnapalan, 2014; Smits et al., 2002) and has a moderate positive effect on self-directed learning ( Koh et al., 2008). Practice-Based Small Groups features a group of providers meeting periodically to review their cases and discuss educational modules. The group members will discuss their practice habits in light of the material they have just reviewed. Talking about how they would apply what they have learned helps increases their confidence in making changes ( Kelly et al., 2007; Overton et al., 2009; Zaher and Ratnapalan, 2012). The Best Practice Conference (BPC) includes many of the lessons learned from these strategies to minimize over-reliance on clinical practice habits and to foster ‘developmental learning’ where the provider makes adaptive changes ( Nilsen et al., 2017). The BPC give residents and faculty members a chance to critically appraise their actions in light of the literature and the conduct of their peers and challenges them to consider modifying their practice.

## Best Practice Conference Protocol

Prior to the Best Practice Conference- The BPC facilitator chooses a common clinical scenario for which there are limited or mixed recommendations in the medical literature. One month before the BPC, the facilitator emails faculty and residents a clinical vignette that embodies the topic and asks which of the listed options would be the provider’s first choice of treatment for this patient, their “Best Practice”. Approximately one week prior to the BPC, the facilitator sends the residents four articles, each advocating a different treatment approach. Four residents are peer-selected to prepare five to ten minute synopses of the articles. Each synopsis focuses on the strengths and weaknesses of one article, the article’s clinical recommendations, and the relative strength of those recommendations. As this was developed in a psychiatry program the BPCs involved treatment of a pregnant woman with psychotic mania and the management of acute psychotic agitation in the Emergency Department. The second scenario included questions on both oral and intramuscular medications.

During the Best Practice Conference- The BPC lasted 90 minutes. Attendees completed a pre-test with the same clinical vignette and questions that were previously distributed by e-mail. After collecting the pre-tests, the facilitator read through the clinical vignette and asked the attendees to announce their “Best Practice” by raising their hand or using an audience response system. The facilitator asked group members to defend their choice based on their clinical experience or knowledge of the literature. Discussants were reminded to focus on their clinical reasoning rather than attacking the rationale for someone else’s choice. As the discussion wound down, the residents presented their synopses of the journal articles. An open-forum discussion of the articles permitted residents and faculty to expound on the strengths or weaknesses of the articles and to relate experiences that confirmed or contradicted the recommendations. Following this discussion the facilitator again led the audience through the clinical vignette. This time they are asked to designate their “Best Practice” choice by “raising your hand proudly and boldly”. Participants were encouraged to look and around and notice how people were voting. After running through all of the available options, the facilitator instructed the participants to find someone who voted differently and “politely convince them that they are wrong”. This variation of peer-based learning led to energetic discussions among the participants. Once the room noise abated, the facilitator reviewed vignette and questions for a third time. The subsequent discussion focused on those who have modified their opinion. Attendees who had changed their “Best Practice” were asked to explain what information had convinced them to alter their recommendation.

Conclusion of the Conference- Participants were given a post-test that contained the same clinical vignette and questions and represented their final “Best Practice” choice. They were asked to predict if the conference would impact their clinical practice and to leave their preferred e-mails so that they could be contacted in the future. Their completing and leaving an e-mail address was considered consent to participant in one further round of questioning. Participants also received a sheet that contained the citations for the references used during the discussion.

Three Month Follow Up- Three months after the BPC, a follow-up survey was sent to the participants. This survey contained the same demographic questions, clinical vignette and questions that participants received prior to and during the conference. Participants were also asked if the BPC had led to a change in their practice patterns.

## Method

The pretest included brief demographic questions about profession and stage of training. The pre-test, post-test, and 3 month follow-up included the clinical vignette and questions that were discussed during the BPC. The pre-and post-tests offered 11 treatment options during the first BPC and 12 options during the second BPC. Participants were asked to pick their “Best Practice” treatment from the list. The post-test also asked participants how likely it was that the BPC would change their practice in regard to that particular patient population (Very Unlikely - Very Likely). The pre-tests and post-tests were given out in packets with random, unique designators so that pre-test and post-test results could be compared while preserving anonymity. The 3 month follow up asked if the BPC had actually led to a change in their treatment of patients. After BPC #1 the question was phrased “Did the Best Practice Conference on Treating Psychotic Mania in a Pregnant Woman change your practice” and the available answers were “None, “Little”, “Some”, or “A Lot”. After BPC#2 this was modified to “The Best Practice Conference changed how I treat a patient with psychotic agitation/aggression” and the available answers were “Disagree”, “Neither Agree nor Disagree” and “Agree”.

The research was approved as exempted research by the Wright State University Institutional Review Board.

## Results

The BPC started at noon resulting in a slight variability in the numbers of responses as providers came and left during the conference. During BPC # 1, thirty-five individuals completed the pre-test including 28 of 34 psychiatry residents, five psychiatry faculty members, and two “other prescribers”. Of the 35 attendees, 33 completed both the pre- and post-tests. Thirteen of 33 respondents (39%) changed their first choice treatment option from pre- to post-test. On the post-test 26 of 34 participants (76%) responded it was “Likely” or “Very Likely” that the conference would change their treatment of patients. Twenty-eight attendees (80%) responded to an e-mail survey three months after the conference. Three of 24 responders indicated that the BPC had changed their practice “A Lot”. (See Table 1)

Best Practice Conference #2 had forty attendees including 31of 33 psychiatry residents, eight psychiatry faculty, and one “other prescriber”. Thirty-nine completed a pre-test and post-test. Nineteen of 39 participants (49%) changed their “Best Practice” for oral medication and 16 (41%) changed their recommendation for intramuscular injections. On the post-test, 18 of 39 (46%) said that it was “Likely” or “Very Likely” that they would change their treatment of a psychotic, agitated patient. Sixteen responded to an e-mail 3 months later, and 4 of them reported that their prescribing practice had changed because of the BPC. (See Table 1)

**Table 1.** Results from the Best Practice Conferences

	BPC #1	BPC # 2	
	Oral Medication	Oral Medication	IM Medication
**Attendees**	35	40	40
**Completed Pre-Test and Post-Test**	33 (94%)	39 (98%)	39 (98%)
**Completed 3-month Follow-up (%)**	28 (80%)	16 (40%)	16 (40%)
**Changed “Best Practice” from pre-test to post-test**	13 (39%)	19 (49%)	16 (41%)
**Post-Test - BPC is “Likely” or Very Likely” to change my practice**	26 of 34 responses (76%)	18 of 39 responses (46%)	
**3-month Follow-up - Did BPC #1 change your practice (A Lot)**	3 of 24 responses (13% of responders; 9% of attendees)		
**3-month Follow-up - BPC #2 changed how I treat patients (Agree)**		4 (25% of responders; 10% of attendees)	

## Discussion

The Best Practice Conference incorporates numerous active techniques to enliven the learning process. The distribution of relevant articles and case vignette creates a ‘flipped classroom’ where conference time is spent discussing rather than teaching basic information. The facilitated group discussions of the providers’ preferred management of the vignette allows for a glimpse into the breadth of treatment diversity within the community and allows for problem-based learning. While the allotted time is inadequate for detailed discussions of the medical literature, participants hear brief summaries of pertinent articles with a review of their strengths and weaknesses that helps promote evidence-based medicine. Peer-based learning occurs as participants attempt to persuade others that theirs is the “Best Practice”. The combination of these strategies ensures lively discussions wherein providers are challenged to consider their own practice in light of the medical literature and the practice patterns of others. It also models critical thinking skills and clinical reasoning for the residents and junior faculty members.

The effectiveness of the Best Practice Conference can be assessed at various depths. Differing clinical experiences and individualized interpretation of the medical literature lead to competing claims for the best treatment, and the active discussion and high level of member participation demonstrated that this was a popular activity. Some of these discussions continued long after the formal Best Practice Conference had ended. An increase in the participants’ medical knowledge can be inferred from the 39 - 49% of attendees who changed their first choice of treatment over the course of the conference. Since all attendees had received the case vignette and questions in advance, any change in their “Best Practice” recommendation came from the interchange of ideas during the BPC. Significant numbers of attenders (46 - 76%) predicted that it was “Likely” or “Very Likely” that the BPC would result in a lasting change in their treatment patterns. The final level of assessment asks if the educational experience actually changed medical practices. Although this was determined by self-report only three months after these conferences, 13-25% of responders reported that the BPC had actually changed their management of patients. A single 1½ hour intervention seemed to modify the practices of at least 9% of the attendees.

Several limitations of this study need to be addressed. Although numerous faculty members were invited, the vast majority of attendees were psychiatry residents. The results may have been different with greater numbers of faculty members. The residents at this institution were somewhat used to having their practice standards challenged in light of medical evidence. Residents and faculty at other facilities may initially be less willing to engage in such emotionally risky conversations. Having respected practitioners provide and receive constructive criticism in an open, non-defensive fashion can create a culture where frank and open discussions of clinical habits and the medical literature are routine. Finally the two conferences combined had less than 80 participants limiting our conclusions. Because of the realities of clinical practice, a few providers entered and left during the session diminishing the impact of the conference and creating inconsistent numbers of responses.

## Conclusion

The Best Practice Conference provides a multifaceted approach to promoting practice based learning and improvement among residents and faculty. Providers can be challenged to consider their practice patterns in light of the medical literature and the experiences of other providers. The use of active learning techniques increases the likelihood that the BPC leads to real change in practice patterns and the preliminary data support this conclusion.

## Take Home Messages

• Continuing medical education should include interactive components such as small group or case-based discussions.
• Peer-based learning techniques ensure that attendees understand and are actively considering the information that has been presented to them.
• Evaluations of continuing medical education should include assessing the likelihood of a change in practice based on the training.
• Continuing medical education should strive to measure actual change in practice based on the training received.

## Notes On Contributors

Dr. Randon S. Welton is the Director of Residency Training in the Department of Psychiatry at Wright State University. He serves as the Chair of the Dayton Area Graduate Medical Education Committee and on the Executive Council of the American Association of Directors of Psychiatry Residency Training. ORCID - https://orcid.org/0000-0002-7062-0665

Dr. Tana Andre is a former psychiatry resident in the Department of Psychiatry at Wright State University. She currently works as a staff psychiatrist at the Montgomery County Emergency Services in Norristown, Pennsylvania.

## References

[ref1] [ACGME] American Council for Graduate Medical Education (2017) Common program requirements. Available at: http://www.acgme.org/Portals/0/PFAssets/ProgramRequirements/CPRs_2017-07-01.pdf( Accessed: 22 Dec 2018).

[ref2] Al-Azri H. and Ratnapalan S. (2014) Problem-based learning in continuing medical education: Review of randomized controlled trials. Canadian Family Physician (Medecin De Famille Canadien). 60(2), pp.157–165. 24522680 PMC3922562

[ref3] Bloom B.S. (2005) Effects of continuing medical education on improving physician clinical care and patient health: A review of systematic reviews. International Journal of Technology Assessment in Health Care. 21, pp.380–385. 10.1017/S026646230505049X 16110718

[ref4] Burke A.E. Benson B. Englander R. Carraccio C. (2014) Domain of competence: practice-based learning and improvement. Academic Pediatrics. 14(2 Suppl), pp.S38–S54. 10.1016/j.acap.2013.11.018 24602636

[ref5] Davis D. (1998) Does CME work? An analysis of the effect of educational activities on physician performance or health outcomes. International Journal of Psychiatry in Medicine. 28(1), pp.21–39. 10.2190/UA3R-JX9W-MHR5-RC81 9617647

[ref6] Kelly D.R. Cunningham D.E. McCalister P. Cassidy J. (2007) Applying evidence in practice through small-group learning: A qualitative exploration of success. Quality in Primary Care. 15, pp.93–99.

[ref7] Koh G.C. Khoo H.E. Wong M.L. and Koh D. (2008) The effects of problem-based learning during medical school on physician competency: A systematic review. Canadian Medical Association Journal (Journal De l’Association Medicale Canadienne). 178(1), pp.34–41. 10.1503/cmaj.070565 PMC2151117 18166729

[ref8] Mansouri M. and Lockyer J. (2007) A meta-analysis of continuing medical education effectiveness. The Journal of Continuing Education in the Health Professions. 27(1), pp.6–15. 10.1002/chp.88 17385735

[ref9] Nilsen P. Neher M. Ellstrom P.E. and Gardner B. (2017) Implementation of evidence-based practice from a learning perspective. Worldviews on Evidence-Based Nursing. 14(3), pp.192–199. 10.1111/wvn.12212 28281328

[ref10] Overton G.K. McCalister P. Kelly D. and Macvicar R. (2009) Practice-based small group learning: How health professionals view their intention to change and the process of implementing change in practice. Medical Teacher. 31(11), pp.514–520. 10.3109/01421590902842425 19909029

[ref11] Smits P.B. Verbeek J.H. and de Buisonje C.D. (2002) Problem based learning in continuing medical education: A review of controlled evaluation studies. British Medical Journal (Clinical Research Ed.). 324, pp.153–156. 10.1136/bmj.324.7330.153 PMC64518 11799034

[ref12] Zaher E. and Ratnapalan S. (2012) Practice-based small group learning programs: Systematic review. Canadian Family Physician (Medecin De Famille Canadien). 58(6), pp.637–642. 22859626 PMC3374683

