# Supplementary material for: The Best Practice Conference: An Interactive Practice-Based Learning Activity for Resident and Faculty Development
Source: MedEdPublish (2016). 2019 Jan 29;8:22. [Version 1] doi: 10.15694/mep.2019.000022.1 (PMC10712537; doi:10.15694/mep.2019.000022.1)
Supplement: Peer review history

# Reviewer report: Reviewer response for version 1

**DOI:** 10.21956/mep.19757.r29654

**Published:** 2019-02-14

**Authors:** Sandra Kemp

**Affiliations:** 1 Curtin Medical School, Curtin University

This review has been migrated. The reviewer awarded 3 stars out of 5 The article describes an interesting design of a learning activity. It will be of interest to readers who wish to create an interactive faculty development session.It was not clear to readers how changing practice was linked with desirable learning. Is there a case for the possibility that change could result in poorer practice? Clarity around change to desired practice versus simply changing from one practice to another would be helpful.When writing about education, I would recommend care in the use of terms that are widely used. For example, Problem-based Learning is not the same as learning about a problem. Terms that describe a set of pedagogical practices (such as PBL) can be distinguished from different pedagogical practices that are commonly used when teaching.I commend the focus in the paper on structuring the learning experience to ensure a high level of interaction during the 'Best Practice Conference'. This provides a clear example for readers.

Reviewer Expertise:

NA

No decision status is available

# Reviewer report: Reviewer response for version 1

**DOI:** 10.21956/mep.19757.r29656

**Published:** 2019-02-08

**Authors:** Balakrishnan Nair

**Affiliations:** 1 Centre for Medical Professional Development and University of Newcastle

This review has been migrated. The reviewer awarded 4 stars out of 5 This article describes an innovative way of teaching/learning . It incorporates PBL and flipped classroom philosophies and reflection as way of learning. Medical practice is never black and white. For the same clinical problem , approaches can vary and learners should understand this and appreciate this in small group debatesHowever they had -only 2 scenarios and the faculty attendance was sub-optimal. I would treat this study as a feasibility project and would love to see a follow up paper with more scenarios and increased faculty participation

Reviewer Expertise:

NA

No decision status is available

# Reviewer report: Reviewer response for version 1

**DOI:** 10.21956/mep.19757.r29653

**Published:** 2019-02-08

**Authors:** Claire MacRae

**Affiliations:** 1 AMEE

This review has been migrated. The reviewer awarded 3 stars out of 5 This was an interesting and well-written article describing how various educational strategies known to encourage deep learning and changes to practice were combined in an innovative way in the ‘Best Practice Conference’. The initiative was designed to promote evidenced-based practice and develop resident’s clinical reasoning skills and should be commended for this. The intervention was clearly described, in sufficient detail to allow others to implement a similar model, and the conclusions and take-home messages are useful to practitioners. One thing that confused me slightly was the success measures of the project. The cases were described as being selected because the literature was limited or provided mixed recommendations. Was the intervention considered a success simply because participants had changed their practice, regardless of what it changed from or to? Or was success measured by changes in practice from a less-desirable to a more-desirable practice? I would like to see this made clearer in any future version of the article. It would also be interesting to know if participants were asked to explain why they had changed their practice – was this based on arguments from the literature? If this wasn’t asked, it might be a nice question for any follow-up study.As a minor point, if the article were to be revised, I would also suggest changing the title so that ‘faculty development’ does not appear as a phrase, as this initiative in not primarily concerned with developing faculty in their educational roles.Aside from these issues, I enjoyed reading the article and would recommend it to anyone looking for new ways to engage peers or junior colleagues in critical thinking and reflection on their practice.

Reviewer Expertise:

NA

No decision status is available

# Reviewer report: Reviewer response for version 1

**DOI:** 10.21956/mep.19757.r29655

**Published:** 2019-01-29

**Authors:** P Ravi Shankar

**Affiliations:** 1 American International Medical University

This review has been migrated. The reviewer awarded 4 stars out of 5 The authors describe an interesting educational intervention among residents and faculty members of Psychiatry. The intervention has been well described and uses many active learning strategies. The limitations of the intervention have also been well described. Only two best practice conference (BPC) sessions have been described. The paper is well-written. The impact of the BPC on practice has been measured at a period of three months after the session through e-mail. Follow up measurements may provide information about the persistence of the change. The manuscript will be of interest to a broad section of medical educators.

Reviewer Expertise:

NA

No decision status is available
